# Early Corticosteroid Therapy May Increase Ventilator-Associated Lower Respiratory Tract Infection in Critically Ill Patients with COVID-19: A Multicenter Retrospective Cohort Study

**DOI:** 10.3390/microorganisms10050984

**Published:** 2022-05-08

**Authors:** Jean-Baptiste Mesland, Eric Carlier, Bruno François, Nicolas Serck, Ludovic Gerard, Charlotte Briat, Michael Piagnerelli, Pierre-François Laterre, on behalf of the COVCORVAP Collaboration Group

**Affiliations:** 1 Critical Care Department, Cliniques Universitaires Saint-Luc, UCLouvain, 1200 Brussels, Belgium; ludovic.gerard@uclouvain.be (L.G.); pierre-francois.laterre@uclouvain.be (P.-F.L.); 2 Intensive Care, CHU-Charleroi Marie Curie, Université Libre de Bruxelles, 6042 Charleroi, Belgium; eric.carlier@chu-charleroi.be (E.C.); michael.piagnerelli@chu-charleroi.be (M.P.); 3 ICU Department and Inserm CIC 1435 & UMR 1092, CHU Dupuytren, 87000 Limoges, France; bruno.francois@chu-limoges.fr (B.F.); charlotte.briat@chu-limoges.fr (C.B.); 4 Unité de Soins Intensifs, Clinique Saint-Pierre, 1340 Ottignies, Belgium; nicolas.serck@cspo.be

**Keywords:** COVID-19, SARS-CoV-2, ventilator-associated lower respiratory tract infection, corticosteroids, acute respiratory distress syndrome

## Abstract

Background: The coronavirus disease 2019 (COVID-19) pandemic has put significant pressure on hospitals and in particular on intensive care units (ICU). Some patients develop acute hypoxemic respiratory failure with profound hypoxia, which likely requires invasive mechanical ventilation during prolonged periods. Corticosteroids have become a cornerstone therapy for patients with severe COVID-19, though only little data are available regarding their potential harms and benefits, especially concerning the risk of a ventilator-associated lower respiratory tract infection (VA-LRTI). Methods: This retrospective multicenter study included patients admitted in four ICUs from Belgium and France for severe COVID-19, who required invasive mechanical ventilation (MV). We compared clinical and demographic variables between patients that received corticosteroids or not, using univariate, multivariate, and Fine and Gray analyses to identify factors influencing VA-LRTI occurrence. Results: From March 2020 to January 2021, 341 patients required MV for acute respiratory failure related to COVID-19, 322 of whom were included in the analysis, with 60.6% of them receiving corticosteroids. The proportion of VA-LRTI was significantly higher in the early corticosteroid group (63.1% vs. 48.8%, *p* = 0.011). Multivariable Fine and Gray modeling considering death and extubation as competing events revealed that the factors independently associated with VA-LRTI occurrence were male gender (adjusted sHR:1.7, *p* = 0.0022) and corticosteroids (adjusted sHR: 1.44, *p* = 0.022). Conclusions: in our multicenter retrospective cohort of COVID-19 patients undergoing MV, early corticosteroid therapy was independently associated with VA-LRTI.

## 1. Introduction

Since December 2019, severe acute respiratory syndrome coronavirus 2 (SARS-CoV-2) has been spreading around the world. In 5–10% of cases, SARS-CoV-2 infection can trigger acute respiratory failure, which requires hospitalization, intensive care unit (ICU) admission, and invasive mechanical ventilation (MV), while being associated with high mortality [1].

Patients undergoing MV for severe SARS-CoV-2 pneumonia are particularly prone to develop ventilator-associated pneumonia (VAP), with an incidence rate as high as 58%, as reported in recent studies [2]. This high VAP incidence could be related to prolonged MV. Indeed, prolonged MV has been associated with an increased risk of VAP, and the duration of MV is longer in patients with acute respiratory distress syndrome (ARDS) related to SARS-CoV-2 infection than in ARDS related to other causes, with a median of 12 days [2].

In addition to the prolonged MV duration, many other factors are likely to increase bacterial superinfection in patients with SARS-CoV-2, including prolonged sedation and neuromuscular blockade [3], use of corticosteroids and immunomodulators, as well as severe lymphopenia [4].

Considering these factors, the link between corticosteroids and VAP is still a matter of debate, given that corticosteroids have been shown to increase [5], decrease [6], or exert no effect [7] on VAP incidence among patients undergoing MV. Besides, early corticosteroids are now recommended as a first-line therapy in patients with severe and critical coronavirus disease 2019 (COVID-19) [8,9], which has led to their current widespread use. Indeed, the RECOVERY trial has demonstrated a reduction in 28-day mortality in COVID-19 patients treated with dexamethasone and requiring supplemental oxygen or MV [10]. A meta-analysis of seven randomized controlled trials generated similar findings with respect to early corticosteroid therapy [11]. The same impact was recently described in patients with classical ARDS [12]. These data have encouraged major medical societies to modify their previous guidelines and strongly recommend using early corticosteroid therapy in patients with severe and critical COVID-19 [8,9]. However, despite the wide use of early corticosteroids among patients with acute respiratory failure related to SARS-CoV-2 infection, the link between corticosteroid usage and VA-LRTI incidence has never been evaluated in large, multicenter cohorts.

The primary objective of this study was to compare, in patients undergoing MV for severe SARS-CoV-2 pneumonia, the occurrence of VA-LRTI between patients who received and those who did not receive early corticosteroid therapy (<7 days since hospital admission). Secondary objectives were to identify potential risk factors for VA-LRTI, along with the microbiology of this events.

## 2. Methods

The appropriate ethics committee (Comité d’Ethique Hospitalo-Facultaire, Saint-Luc, UCL N° 2021/15JAN/016) approved the reporting of our study results. Informed consent from the patients or their next of kin was waived owing to the retrospective nature of the analysis.

### 2.1. Patients

This was a multicenter retrospective study. Patients admitted between March 2020 and January 2021 to four ICUs in Belgium and France were included in the analysis, based on the following criteria: age equal or above 18 years; diagnosis of SARS-CoV-2 pneumonia based on a positive polymerase chain reaction (PCR) test; MV requirement for more than 48 h.

The patient management was not protocolized yet was rather similar among centers. Patients were admitted to the ICU in the event of severe hypoxemic respiratory failure (persisting hypoxemia despite O_2_ > 15 L/min), hemodynamic instability, or altered consciousness. Initial management consisted of either high-flow nasal canula (HFNC) or non-invasive ventilation (NIV). Escalation to invasive MV was only performed in the presence of refractory hypoxemia despite optimized HFNC or NIV, poor clinical tolerance, or altered consciousness. Management of ARDS and VAP prevention care bundle were conducted in line with current guidelines [13,14]. Prevention care bundle included hand hygiene, head of bed elevation (30–45°), oral care, reduced exposure to MV with spontaneous breathing trial, daily sedation interruption whenever possible and targeted sedation by Richmond Agitation Sedation Scale (RASS), deep vein thrombosis prophylaxis, closed suction catheters, monitoring and control of endotracheal cuff pressure (not automated), enteral route feeding, as well as physiotherapy [14]. According to local practices, antimicrobial therapy was only initiated if VAP was suspected, as based on clinical, radiological, and biological criteria.

Corticosteroids were used as standard of care from June 2020 onwards, after preliminary results from the REVOVERY trial were released. Dexamethasone and methylprednisolone were employed at a dosage of 6 mg and between 1 mg/kg and 2 mg/kg for 10 days, respectively.

### 2.2. Data

Data were collected from electronic medical systems and patients’ medical charts and recorded into a case report form (CRF). These data included demographics and baseline characteristics (age at admission, gender, body mass index, as well as comorbidities including immune status), COVID-19-specific treatments, respiratory parameters at ICU admission and at the time of intubation (PaO_2_/FiO_2_ ratio), and adjuvant therapies such as prone positioning, vasopressors, and SOFA scores were calculated at ICU admission. Study Day 0 corresponded to the first day of invasive MV. The occurrence of VA-LRTI and bloodstream infection (BSI) was carefully recorded, alongside microbiological documentation and antibiotic therapy, over the first 30 MV days. Patients were followed-up until hospital discharge, and whether they were alive or dead was recorded at both ICU discharge and Day 28.

### 2.3. Definitions

The COVID-19 cases were defined as the combination of a positive RT-PCR for SARS-CoV-2 in a nasopharyngeal swab or in broncho-alveolar lavage specimens [15] and consistent abnormalities in chest X-rays or computed tomography [16]. Immunosuppression was defined as chronic immunosuppressive therapy including corticosteroids, human immunodeficiency virus infection, or malignant hemopathy.

Early corticosteroid therapy was defined as corticosteroid therapy (dexamethasone or methylprednisolone ≥ 1 mg/kg) within the first 7 days of hospital admission [17].

We defined VA-LRTI [18] upon 48 h of MV. At least one of the following symptoms was required: fever (>38 °C) without any other cause, leukopenia (<4000/mm^3^), leukocytosis (>12,000/mm^3^), and at least one of the following respiratory signs, including new onset of purulent sputum, change in sputum features (color, odor, quantity, or consistency), or gas exchange worsening (e.g., O_2_ desaturation, increased oxygen requirements, or increased ventilation demand). Microbiological sampling was mandatory to establish VA-LRTI diagnosis, which could either consist of a noninvasive sampling with semiquantitative cultures or an invasive sampling with quantitative semiquantitative cultures. Invasive respiratory sampling comprised bronchoscopic techniques (i.e., bronchoalveolar lavage (BAL), protected specimen brush) and blind bronchial sampling (i.e., mini-BAL), with non-invasive respiratory sampling referring to endotracheal aspiration [19]. Early VA-LRTI was defined as that occurring within the first 5 days of intubation and late VA-LRTI as that occurring at 5 days or after 5 days of intubation [19].

### 2.4. Statistical Analysis

Statistical analyses were performed using the SPSS 21 software (SPSS software (IBM Corp. 2011. IBM SPSS Statistics for Windows, Version 21.0. Armonk, NY, USA: IBM Corp.)) and R 4.0.5 (package survival), with graphs drawn via GraphPad Prism 8.0 (GraphPad Software, La Jolla, CA, USA). Values were expressed as mean (+/− standard deviation) for continuous variables and counts (per percent of group) for qualitative variables. The data underwent Kolmogorov–Smirnov normality and Bartlett’s testing for homogeneity of variance. We compared clinical and demographic variables between patients receiving and those not receiving corticosteroids (with and without VA-LRTI), using the Chi-squared test or Fisher’s exact as appropriate and unpaired *t*-testing for quantitative data, respectively. Comparison of VA-LRTI incidence between study groups was performed by means of Fine and Gray models, with extubation and death considered as competing events. In order to identify factors influencing VA-LRTI occurrence, taking into account competing events such as extubation and death, sub-hazard ratios were calculated by means of Fine and Gray models [20]. In addition, comparisons were adjusted for confounders suspectedly associated with VA-LRTI, such as age, immunosuppression, recent antibiotic therapy, and ARDS severity, based on multivariable Fine and Gray models. In order to compare outcomes between study groups, cause-specific hazard for duration of mechanical ventilation and ICU stay was calculated by means of cause-specific Cox hazard models, with mortality considered a competing event. All tests were two-sided, with a significance level set at 0.05.

## 3. Results

From March 2020 to January 2021, 513 patients were admitted to ICUs for severe SARS-CoV-2 pneumonia, with 341 of them requiring MV (Supplementary Material Figure S1). Data were missing for six patients, and 13 were ventilated for less than 48 h. These patients were excluded from the analysis. Among the 322 patients included in the analysis, 195 received corticosteroids (60.6%) and 127 (39.4%) did not (Table 1).

The patient characteristics at baseline, in addition to adjuvant therapies, respiratory variables, and outcomes, are summarized in Table 1 and Table 2. Baseline characteristics were similar between groups, except for immunosuppression being more prevalent in the corticosteroid group (12.3% vs. 4.7%, *p* = 0.024). PaO2/FiO2 ratio upon ICU admission was lower in the corticosteroid group (86.8 mmHg +/− 42 vs. 104.2 mmHg +/− 54, *p* = 0.014), as was a higher proportion of severe ARDS (73.3% vs. 59.8%, *p* = 0.011) herein. Dexamethasone was employed in 174/195 (89.2%) patients receiving corticosteroid therapy. Corticosteroids were initiated within 3 days prior to intubation (+/−4.5 days) on average and were continued for 9.5 days (+/−2.4 days). A lower proportion of patients received hydroxychloroquine (2.6% vs. 46.4%, *p* < 0.0001) or azithromycin (2.1% vs. 11.8%, *p* = 0.0003) in the corticosteroid group. Similarly, prior antibiotic treatment before ICU admission was less commonly employed in the corticosteroid group (35.8% vs. 57.5%, *p* = 0.0002).

Clinical outcomes did not differ according to corticosteroid treatment, including ICU length of stay, duration of ventilation, and ventilatory-free days at Day 28, whereas there was a non-significant trend towards higher ICU mortality (51.3% vs. 40,2%, *p* = 0.051) among patients receiving corticosteroids. The VA-LRTI proportion was significantly higher in the corticosteroid group (63.1% vs. 48.8%, *p* = 0.011), reflected in both early VA-LRTI (11.8% in the non-corticosteroid group vs. 18.5% in the corticosteroid group) and late VA-LRTI (37% in the non-corticosteroid group vs. 44.6% in the corticosteroid group) subpopulations. Time to the first VA-LRTI episode was 7.4 days +/− 4.6 days vs. 8.9 +/− 5.7 days (*p* = 0.06) in the corticosteroid vs. non-corticosteroid group, respectively. We performed a posteriori a power calculation to reject the null hypothesis of equal proportion (with a significance level of 0.05), based on the observed difference of VA-LRTI between groups. With a group sample size of 322, we achieved a 95% power to reject the null hypothesis.

**Figure 1:**
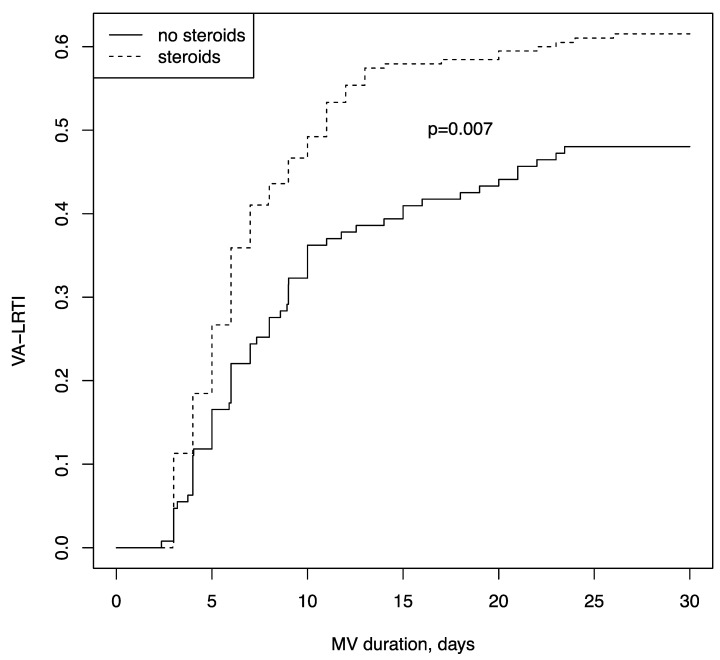
Cumulative incidence of ventilatory-associated lower respiratory tract infection within 30 days of mechanical ventilation, using a Fine and Gray model, considering extubation or death within 30 days as competing events. VA-LRTI: ventilator-associated respiratory tract infection.

In addition, we compared the incidence of the first episode of VA-LRTI between patients with and without corticosteroids using Fine and Gray competing risk models, while considering the competing risks of death and extubation. As shown in Figure 1 and Supplementary Material Figure S2, VA-LRTI incidence was still significantly higher in the corticosteroids group (*p* = 0.0077). The same results as for VA-LRTI occurrence (66% vs. 48%, *p* = 0.0025) were observed after excluding the patients in whom steroids were started after intubation, in order to avoid the risk of immortal time bias.

Multivariable Fine and Gray models were additionally employed to identify factors that were independently associated with VA-LRTI occurrence within the first 30 days of MV, while considering the competing risks of death and extubation. Corticosteroid treatment and male gender were both associated with an increased hazard for VA-LRTI (unadjusted sHR 1.61, [1.17–2.02], *p* = 0.0028, and unadjusted sHR = 1.74 [1.24–2.44], *p* = 0.0013, respectively) (Table 3). This association remained significant after adjusting for confounding factors (adjusted sHR = 1.45 [1.05–199], *p* = 0.022 and adjusted sHR = 1.7 [1.21–2.39], *p* = 0.002, respectively). Using multivariable cause-specific hazard models, corticosteroids were associated with significantly decreased hazards of extubation (HR 0.72 [0.53–0.98], *p* = 0.038) and of ICU discharge alive (HR 0.73 [0.53–0.99], *p* = 0.048) (both with mortality considered as a competing event) (Supplementary Material Tables S1 and S2).

As there was imbalance between groups regarding prior immunosuppression, IL-6 interfering therapies, and extra-corporeal membrane oxygenation (ECMO) use, post hoc analyses were made after excluding patients with prior immunosuppression, those receiving IL-6 interfering therapies, or patients undergoing ECMO, with consistent results regarding VA-LRTI occurrence (65% in the corticosteroid group vs. 48.3%, *p* = 0.0048; 63.2% vs. 48.8% *p* = 0.011 and 60.1% vs. 47.8%, *p* = 0.042, respectively). The main causes of immunosuppression are summarized in Supplementary Material Table S3.

Baseline characteristics, adjunctive treatments, and outcomes between patients with or without VA-LRTI or between patients from the first wave as opposed to patients from the second/third wave have been summarized in Supplementary Material Tables S4–S7.

During the first 30 days of ventilation, overall, 227 VA-LRTI episodes were documented in the 185 patients with VA-LRTI during this period. Of these, 19,8% were considered as early VA-LRTI (<5 days from intubation) and 80.2% as late VA-LRTI (≥5 days from intubation). The microorganisms responsible for VA-LRTI are listed in Table 4. Overall, 73.2% of isolates for VA-LRTI were *Gram-negative bacilli*, 26.2% *Gram-positive cocci*, and 0.6% *Gram-negative cocci*. The predominant pathogen was *Staphylococcus aureus* in 22.4% of VA-LRTI, followed by *Pseudomonas aeruginosa* (15.1%) and *Escherichia Coli* (13.2%).

## 4. Discussion

Based on this multicenter retrospective cohort of mechanically ventilated patients with severe SARS-CoV-2 pneumonia, we have demonstrated that VA-LRTIs were rather common and that early corticosteroid therapy was associated with an increased VA-LRTI incidence within the first 30 days of MV initiation.

The VA-LRTI occurrence was estimated at 57.5% in our cohort, which is consistent with results recently reported by Rouzé (50.5% of VA-LRTI incidence) [21], as well as a recent meta-analysis [22] that reported a pooled estimated VAP occurrence of 45.4%. Compared to classical ARDS or other viral infection requiring MV, COVID-19 has been described as an independent risk factor for VAP [21]. As already outlined in the paper’s Introduction section, several pathophysiological features of severe SARS-CoV-2 pneumonia likely explain this high VA-LRTI incidence.

In our cohort, patients who developed a VA-LRTI exhibited increased ICU length of stay, duration of MV, and decreased 28-day ventilatory-free days, which is perfectly in line with recently reported observations concerning COVID-19 and non-COVID-19 patients [19,22]. However, in our cohort, no significant association between VA-LRTI and Day-28 or ICU mortality was observed. Indeed, the relationship between VA-LRTI and mortality in COVID-19 patients is still fiercely debated in the scientific community. Nseir et al. revealed a significant association between VA-LRTI occurrence and 28-day mortality in their cohort involving 568 COVID-19 patients undergoing MV [23], whereas the opposite was recently shown pertaining to the findings of a meta-analysis [22].

The main observation arising from our study was that early corticosteroid therapy was independently associated with an increased VA-LRTI incidence in patients undergoing MV for severe COVID-19. To our knowledge, with 322 patients included and 195 (60.5%) receiving corticosteroids, our cohort is the largest patient population published to date with the aim to investigate the effects of corticosteroids on the risk of VA-LRTI. Prior evidence regarding the influence of corticosteroids on the occurrence of VA-LRTI is rather scarce, but recent studies have generated consistent results. Recently, an increased VAP incidence associated with corticosteroid treatment has been reported in a cohort involving 250 COVID-19 patients [24]. However, a high proportion (60%) of these cohort patients were treated with tocilizumab. Therefore, caution is required when extrapolating these results to different patient populations. In our cohort, the use of IL-6- and IL-1-modulating therapies turned out to be rather low (3.7%), with the same results regarding VA-LRTI incidence found after excluding these patients from the analysis. Furthermore, dexamethasone was reported to be associated with increased infectious event rates in a 100-patient population, yet with a high prevalence of underlying immune defects (34%) [25]. In our cohort, there was a low proportion of patients with prior immunosuppression (9.3%), yet with a significant imbalance between groups, given that significantly more patients with prior immunosuppression were among the corticosteroid-treated group (12.3% vs. 4.7%, *p* = 0.024). Nevertheless, similar results were found when these patients were excluded in a post hoc analysis; in multivariate analysis, immunosuppression turned out not to be associated with an increased VA-LRTI risk. Furthermore, recent data are rather conflicting concerning the link between prior immunosuppression and VA-LRTI risk. A planned analysis of the prospective multinational TAVeM database involving 2960 patients (without COVID-19), with 663 (22%) among whom displayed known immunosuppression, reported a decreased VA-LRTI incidence in immunocompromised patients compared to non-immunocompromised patients [18]. Conversely, no association between immunosuppression and VAP occurrence was found in a cohort of 188 patients undergoing MV for severe COVID-19 [26].

As shown in Figure 1, the difference regarding VA-LRTI incidence between patients with or without corticosteroids was likely to occur early on or within the 10 days of MV. However, many patients were no longer included in the final analysis, owing to either death or extubation; thus, the number patients still “at risk” (free of VA-LRTI, alive, and still under MV) at later time points was rather low. Therefore, the lack of difference between groups at later time points may be due to the fact that corticosteroid impact on VA-LRTI risks fades away with time; another possibility may be that the number of patients at risk at later time points was not sufficient to retrieve a between-group difference.

In our data, we found a non-significant trend towards a higher 28-day mortality (or in ICU mortality) between patients who received early corticosteroid therapy and those who did not. This observation is in contrast with the RECOVERY trial results [10], which demonstrated a decreased mortality at Day 28 in patients who received 10 days of dexamethasone, the greatest effect being observed in patients under MV and those with symptoms lasting for more than 7 days. The reasons for this discrepancy between our data and other studies concerning the impact of corticosteroids on outcome are not yet clearly understood. In our cohort, ICU mortality turned out to be high (47.7%), thereby reflecting our usual management strategy that consists of only performing MV in those patients exhibiting the most severe disease, who are refractory to either HFNC or NIV. This selection bias towards the sickest patients could possibly explain the difference in the corticosteroid impact on outcomes compared to recent prospective trials. Of note, corticosteroids have recently been shown to exert less beneficial effects in certain patient subgroups, including elderly patients [27] and those with a hypo-inflammatory phenotype [28,29]. As expressed by De Backer et al., the beneficial effects on the Day-28 mortality endpoint, relatively short in duration, may not necessarily translate into longer-term benefits [30]. Further studies are thus warranted to confirm the mortality benefits with respect to a longer term than the Day-28 outcome.

Besides corticosteroid therapy, male gender was independently associated with VAP. Blonz et al. found the same association in univariate and multivariate analyses between male gender and VAP occurrence in COVID-19 ventilated patients [26]. Similar findings have previously been described in COVID-19-unrelated ARDS patients and multiple trauma patients on MV [31]. Gender predominance has been described in MERS and SARS-CoV-1 too [32,33]. This link may be accounted for by several factors such as gender-based differences in co-morbidities, gender-based socio-cultural and behavioral differences, but also by fundamental differences in the innate and adaptive immune systems [34].

The strengths of this study comprise the multicenter study design, in addition to the well-balanced groups regarding corticosteroid treatment. Indeed, the limited use of other immunomodulatory drugs allowed us to better focus on the corticosteroid impact. Moreover, we were able to analyze the data of nearly all the patients who met the inclusion criteria.

The limitations of this study must also be acknowledged. First, our study used a retrospective and observational study design. Taking this into account, it must be stressed that our results could potentially have been influenced by multiple biases, including in particular “immortal time bias” [35]. Nevertheless, the corticosteroid initiation preceded intubation in a large majority of patients, and the delay between intubation and steroid initiation was very short in the few patients in whom steroids were initiated after intubation. Therefore, the influence of an immortal time bias, if any, would be rather limited. Moreover, when our analysis was restricted to the sole patients in whom corticosteroids were initiated before intubation, similar results concerning the influence of corticosteroids on VA-LRTI occurrence were found. Second, we included patients between March 2020 and January 2021, thus pertaining to three different waves. Most patients who received corticosteroids were hospitalized after June 2020 (release of the results of the RECOVERY trial), during the second and third waves. As COVID-19 was previously unknown, patient selection criteria for ICU admission, adjunctive treatments, and ICU management had evolved during this period. This may thus constitute a selection bias. Patients who received corticosteroids exhibited higher disease severity at baseline (probably related to evolving ICU admission criteria) and were less likely to receive hydroxychloroquine, azithromycin, or prior antibiotic treatments (due to the evolution of available evidence concerning the efficacy of these treatments). However, it is unclear how these differences between groups could have influenced the observed difference in terms of VA-LRTI occurrence. The higher disease severity at baseline in the corticosteroid group was not associated with longer MV exposure. Notably, ARDS severity per se was not shown to be an independent predictor of VA-LRTI risk [36]. Hydroxychloroquine and azithromycin have been shown to be ineffective in managing COVID-19 [37,38,39], and they are thus unlikely to have influenced our results. Based on recent results, prior exposure to antibiotic therapy was reported as unlikely to influence VA-LRTI occurrence among patients with COVID-19 [26], yet such treatment may well induce the occurrence of multi-drug resistant organisms. Moreover, the association between corticosteroid and VA-LRTI remained significant after adjusting for these pre-specified confounders in a multivariable Fine and Gray model. However, other unmeasured confounders, known as possible risk factors for VA-LRTI (such as sedation, glycemic control, VAP bundle prevention compliance, and use of prone positioning) could also have influenced the study results. Third, the study was conducted in Europe only, namely in Belgium and France, and the results may thus not be extrapolated across the remaining world. Fourth, we have not used quantitative methods, as an invasive technique was not mandatory for VA-LRTI diagnosis. However, a Cochrane review revealed no significant differences in terms of mortality, length of ICU stay, duration of mechanical ventilation, and rate of antibiotic change between qualitative culture of noninvasive samples and quantitative culture of invasive samples [40], and similar methods were employed between corticosteroid and non-corticosteroid groups. Fifth, we limited our analysis to VA-LRTI occurrence within the first 30 days of MV.

Further studies are now warranted to further confirm the potential harms and benefits of early corticosteroid therapy in COVID-19 patients undergoing MV and to better identify patients in whom corticosteroids may turn out to be harmful.

## 5. Conclusions

To conclude, in this multicentric retrospective cohort involving mechanically ventilated COVID-19 patients, treatment with early corticosteroid therapy was independently associated with VA-LRTI occurrence.

## Figures and Tables

**Table 1:** Characteristics between patients receiving early corticosteroid therapy and those who did not.

	All Population (*n* = 322)	No Steroid (*n* = 127)	Steroid (*n* = 195)	*p* Value
**Baseline characteristics**				
Age, years (+/−SD)	64.8 (+/−10.4)	65 (+/−10)	64.6 (+/−10.7)	0.71
Male (%)	219/322 (68)	90/127 (70.9)	129/195 (66.2)	0.38
BMI (+/−SD)	29.7 (+/−6.2)	29 (+/−4.8)	30.2 (+/−6.9)	0.081
Hypertension (%)	204/322 (63.3)	78/127 (61.4)	126/195 (64.6)	0.62
Diabetes mellitus (%)	132/322 (41)	46/127 (36.2)	86/195 (44.1)	0.18
Cardiomyopathy (%)	49/322 (15.2)	19/127 (14.6)	30/195 (15.4)	0.94
Chronic kidney disease (%)	33/322 (10.2)	8/127 (6.3)	25/195 (12.8)	0.062
Immunosuppression (%)	30/322 (9.3)	6/127 (4.7)	24/195 (12.3)	0.024
COPD (%)	28/322 (8.7)	9/127 (7.1)	19/195 (9.74)	0.42
Neoplasia <2 years (%)	21/322 (6.5)	5/127 (4)	16/195 (8.2)	0.13
**Admission characteristics**				
SOFA (+/−SD)	5.6 (+/−2.4)	5.4 (+/−2.4)	5.7 (+/−2.5)	0.26
APACHE II (+/−SD)	16 (+/−5.5)	16.1 (+/−4.5)	15.9 (+/−5.8)	0.73
PaO_2_/FiO_2_, mmHg (+/−SD)	93.7 (+/−47.8)	104.2 (+/−54)	86.8 (+/−42)	0.0014
Mild ARDS (PaO_2_/FiO_2_ < 300 mmHg)	10/322 (3.1)	6/127 (4.7)	4/195 (2.1)	0.17
Moderate ARDS (PaO_2_/FiO_2_ < 200 mmHg)	89/322 (27.6)	43/127 (33.9)	46/195 (23.6)	0.044
Severe ARDS (PaO_2_/FiO_2_ < 100 mmHg)	219/322 (68)	76/127 (59.8)	143/195 (73.3)	0.011
Ferritin, mcg/L (+/−SD), (*n* = 165)	2041 (+/−1794)	2051 (+/−1808)	2035 (+/−1794)	0.95
CRP, mg/L (+/−SD)	172.9 (+/−101.5)	186.1 (+/−103.5)	165,2 (+/−99.7)	0.082
Lymphocytes, /mcL (+/−SD)	840 (+/−996)	780 (+/−427)	878 (+/−1231)	0.39
Shock (%)	53/322 (16.4)	18/127 (14.2)	35/195 (18)	0.37
Prior antibiotic treatment (%)	143/322 (44.7)	73/127 (57.5)	70/195 (35.8)	0.0002
**Treatment**				
Hydroxychloroquine (%)	64/322 (19.9)	59/127 (46.4)	5/195 (2.6)	<0.0001
Azythromycin (%)	19/322 (5.9)	15/127 (11.8)	4/195 (2.1)	0.0003
Remdesivir (%)	13/322 (4)	6/127 (4.7)	7/195 (3.6)	0.61
Immunomodulating therapies, IL6 and IL1 antagonist (%)	12/322 (3.7)	2/127 (1.6)	10/195 (5.1)	0.10
ECMO (%)	44/322 (13.7)	12/127 (9.5)	32/195 (16.4)	0.076
Prone positionning (%)	271/322 (84.2)	108/127 (86.4)	163/195 (83.6)	0.78
Sedation, days (+/−SD)	14.4 (+/−13.2)	12.9 (+/−9.9)	15.3 (+/−14.9)	0.11
Hospital admission to intubation, days (+/−SD)	4.2 (+/−5.8)	4.2 (+/−6.6)	4.2 (+/−5.2)	0.96

ICU: intensive care unit, BMI: body mass index; COPD: chronic obstructive pulmonary disease; SOFA: sequential organ failure assessment; APACHE II: acute physiology and chronic health evaluation II; ARDS: acute respiratory distress syndrome; ECMO: extra-corporeal membrane oxygenation.

**Table 2:** Patients’ outcomes between patients receiving early corticosteroid therapy and those who did not.

	All Population (*n* = 322)	No Steroid (*n* = 127)	Steroid (*n* = 195)	*p* Value
ICU mortality (%)	151/322 (46.9)	51/127 (40.2)	100/195 (51.3)	0.051
ICU Day-28 mortality (%)	120/322 (37.3)	40/127 (31.5)	80/195 (41)	0.084
ICU length of stay, days (+/−SD)	23.1 (+/−20.9)	22.9 (+/−15.9)	23.2 (+/−23.7)	0.89
Duration of ventilation, days (+/−SD)	18.3 (+/−17)	17.9 (+/−14)	18.5 (+/−18.7)	0.73
Ventilatory-free day D28 (+/−SD)	6.8 (+/−8.7)	7.6 (+/−8.3)	6.3 (+/−9)	0.19
Bloodstream infection (%)	73/322 (22.6)	23/127 (18.1)	50/195 (25.6)	0.11
Ventilator-associated lower respiratory tract infection (%)	185/322 (57.5)	62/127 (48.8)	123/195 (63.1)	0.011

ICU: intensive care unit.

**Table 3:** Variables associated with a first episode of VA-LRTI in 322 patients with severe COVID-19, using multivariable Fine and Gray models, considering death and extubation as competing events. Adjusted sub-hazard ratios are adjusted for prespecified confounders suspected to be associated with VA-LRTI, including age, immunosuppression, recent antibiotic treatment, and ARDS severity.

	**Multivariable Fine and Gray Model**					
**Variables**	Unadjusted Sub-hazard ratio	IC 95	*p*	Adjusted Sub-hazard ratio	IC 95	*p*
**Corticosteroids**	1.61	1.17–2.02	0.003	1.44	1.05–1.98	0.022
**Male Sex**	1.74	1.24–2.44	0.002	1.70	1.21–2.39	0.0022

**Table 4:** Microorganisms responsible for ventilator-associated lower respiratory tract infection within the first 30 days of MV in the early corticosteroid group vs. non-corticosteroid group.

Microorganisms	Non CS Group	% Isolate	% VA-LRTI	CS Group	% Isolate	% VA-LRTI	Total	% Isolate	% VA-LRTI
***Gram-positive cocci***	***25***	***25.8***	***34.7***	***58***	***26.4***	***37.4***	***83***	***26.2***	***36.6***
MSSA	20	20.6	27.8	43	19.4	26.9	63	19.9	27.8
MRSA	1	1	1.4	7	2.0	2.7	8	2.5	3.5
Streptococcus pneumoniae	2	2.1	2.8	7	2.0	2.7	9	2.8	4.0
Streptococcus agalactiae	0	-	-	1			1	0.3	0.4
Streptcoccus constellatus	1	1	1.4	0	-	-	1	0.3	0.4
Streptococcus dysgalactiae	1	1	1.4	0	-	-	1	0.3	0.4
***Gram-negative bacilli***	***72***	***74.2***	***100***	***160***	***72.7***	***103.2***	***232***	***73.2***	***102.2***
Pseudomonas aeruginosa	17	17.5	23.6	31	14.1	20	48	15.1	21.1
Escherichia coli	16	16.5	22.2	26	11.8	16.8	42	13.2	18.5
Enterobacter cloacae	5	5.2	6.9	16	7.3	10.3	21	6.6	9.3
Klebsiella pneumoniae	4	4.1	5.6	17	7.7	11	21	6.6	9.3
Citrobacter koresi	4	4.1	5.6	11	5	7.1	15	4.7	6.6
Proteus mirabilis	4	4.1	5.6	8	3.6	5.2	12	3.8	5.3
Serratia marcescens	4	4.1	5.6	7	3.2	4.5	11	3.5	4.8
Moraxella catarhalis	0	-	-	2	0.9	1.3	2	0.6	0.9
Klebsiella aerogenes	2	2	2.8	13	5.9	8.4	15	4.7	6.6
Klebsiella oxytoca	4	4.1	5.6	2	0.9	1.3	6	1.9	2.6
Stenotrophomonas maltophilia	0	-	-	5	2.3	3.2	5	1.6	2.2
Haemophilius influenzae	7	7.2	9.7	14	6.4	9	21	6.6	9.3
Citrobacter freundii	1	1	1.4	2	0.9	1.3	3	0.9	1.3
Morganella morganii	0	-	-	3	1.4	1.9	3	0.9	1.3
Klebsiella varicola	0	-	-	2	0.9	1.3	2	0.6	0.9
Hafnia alvei	1	1	1.4	0	-	-	1	0.3	0.4
Chryseobacterium indologenes	1	1	1.4	0	-	-	1	0.3	0.4
Proteus vulgaris	1	1	1.4	0	-	-	1	0.3	0.4
Achromobacter	1	1	1.4	0	-	-	1	0.3	0.4
Raoultella ornithinolytica	0	-	-	1	0.5	0.6	1	0.3	0.4
***Gram-negative cocci***	***0***	-	-	***2***	***0.9***	***1.3***	***2***	***0.6***	***0.9***
Neisseria meningitidis	0	-	-	2	0.9	1.3	2	0.6	0.9

CS: corticosteroid; VA-LRTI: ventilatory-associated lower respiratory tract infection.

## Data Availability

The datasets used and/or analyzed during the current study are available from the corresponding author on reasonable request.

## Supplementary Materials

The following supporting information can be downloaded at https://www.mdpi.com/article/10.3390/microorganisms10050984/s1, Figure S1: Study cohort flow chart. Figure S2: Cumulative incidence of ventilatory-associated lower respiratory tract infection within 30 days of mechanical ventilation (black), using a Fine and Gray model, considering extubation (green) or death (red) within 30 days as competing events. Table S1: Variables associated with successful extubation in 322 patients with severe COVID-19, using a cause-specific hazard model, with mortality considered as a competing event. Table S2: Variables associated with discharge alive from the ICU in 322 patients with severe COVID-19, using a cause-specific hazard model, with mortality considered as a competing event. Table S3: Characteristics (pathology and medication) of the thirty patients considered as immunosuppressed. Table S4: Patients characteristics between those developing VA-LRTI or not. Table S5: Patients’ outcomes between those developing VA-LRTI or not. Table S6: Patients characteristics between first vs. second and third waves. Table S7: Patients’ outcomes between first vs. second and third waves.

## Abbreviations

SARS-CoV-2	Severe acute respiratory syndrome coronavirus 2
COVID-19	Coronavirus disease 2019
ICU	Intensive care unit
VA-LRTI	Ventilator-associated lower respiratory tract infection
VAP	Ventilator-associated pneumonia
VAT	Ventilator-associated tracheobronchitis
VFD	Ventilator-free days
ICU	Intensive care unit
LOS	Length of stay
MV	Mechanical ventilation
HFNC	High-flow nasal cannula
NIV	Non-invasive ventilation
ARDS	Acute respiratory distress syndrome
ECMO	Extra-corporeal membrane oxygenation
BSI	Bloodstream infection
