# Supplementary material for: Early Corticosteroid Therapy May Increase Ventilator-Associated Lower Respiratory Tract Infection in Critically Ill Patients with COVID-19: A Multicenter Retrospective Cohort Study
Source: Microorganisms. 2022 May 8;10(5):984. doi: 10.3390/microorganisms10050984 (PMC9146632; doi:10.3390/microorganisms10050984)
Supplement: Supplementary file 1 [file microorganisms-10-00984-s001.zip › microorganisms-1678762-supplementary.pdf]

Supplementary Materials

Figure S1: Study cohort flow chart.

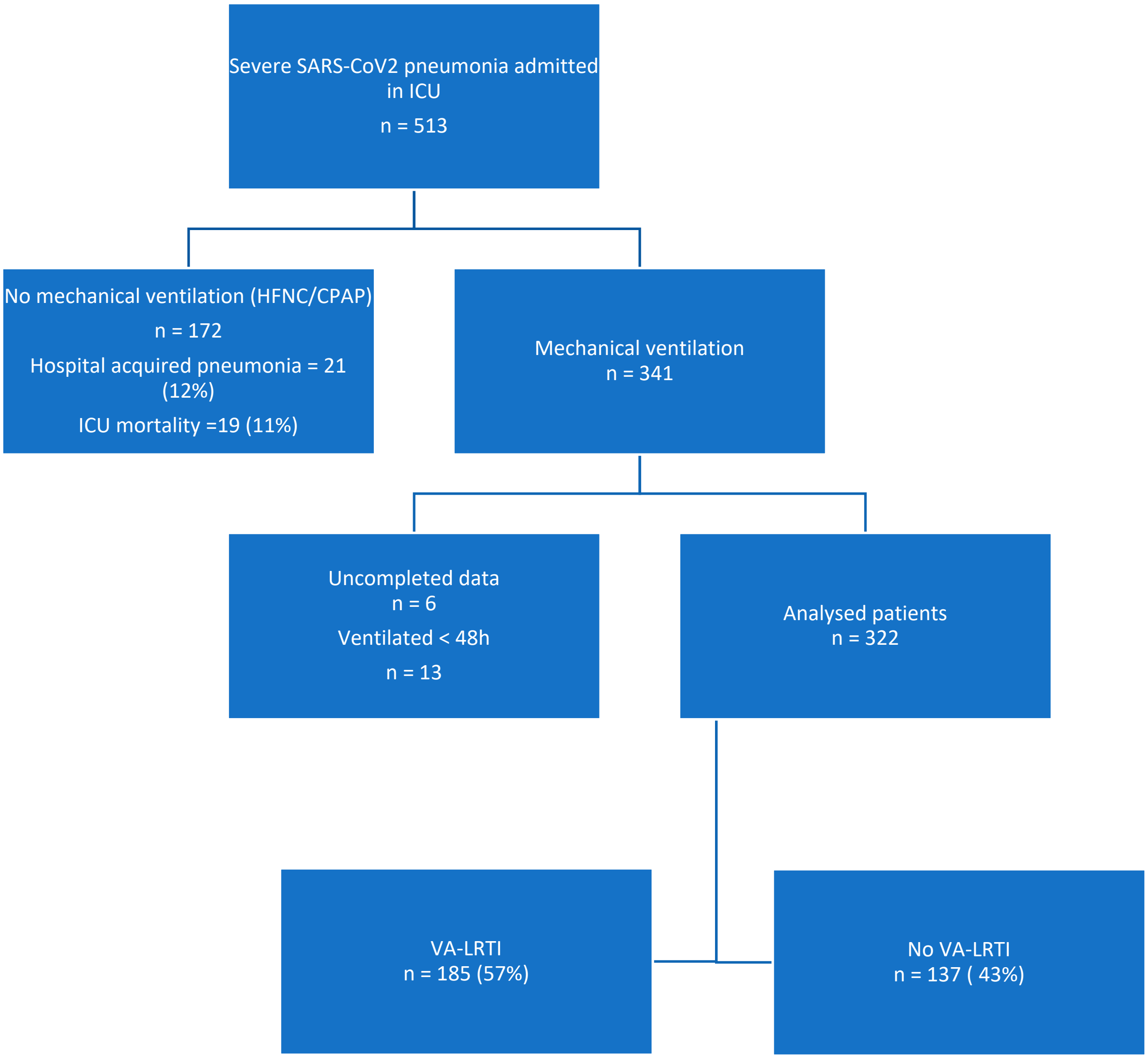

ICU: intensive care unit; HFNC: high-flow nasal cannula; VA-LRTI: ventilator-associated lower respiratory tract infection.

Figure S2: Cumulative incidence of ventilatory-associated lower respiratory tract infection within 30 days of mechanical ventilation (black), using a Fine and Gray model, considering extubation (green) or death (red) within 30 days as competing events.

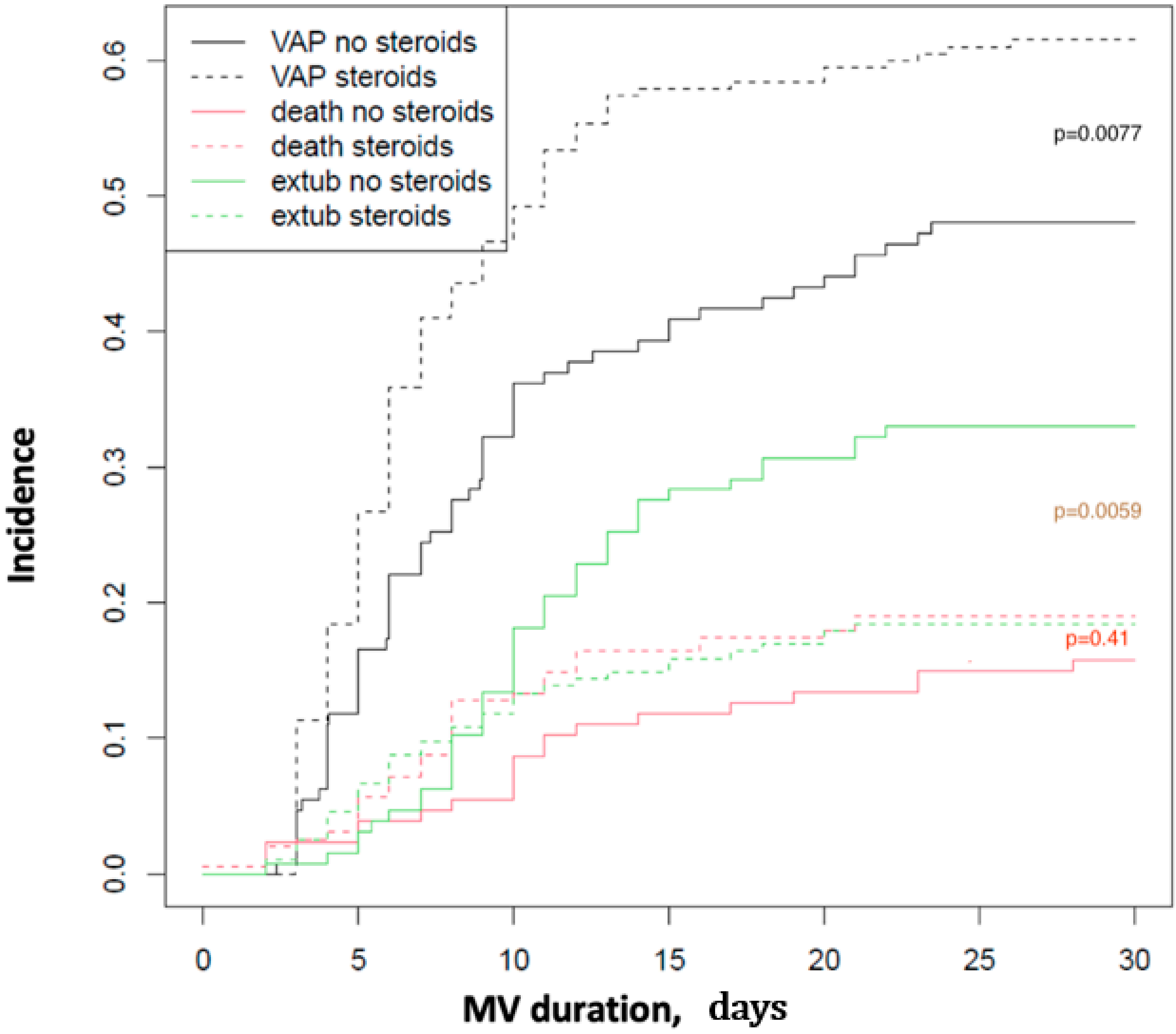

VA-LRTI: ventilator-associated respiratory tract infection; MV: mechanical ventilation.

Table S1: Variables associated with successful extubation in 322 patients with severe COVID-19, using a cause-specific hazard model, with mortality considered as a competing event.

	**Multivariate**		
**Variables**	Adjusted Hazard Ratio	IC 95	p
**Corticosteroids**	0.72	0.53–0.98	**0.038**
**Male Sex**	0.75	0.53–1.04	**0.09**
**Age**	0.97	0.95–0.99	**0.0004**
Chronic Kidney Disease	1.51	0.83–2.75	0.17
Hypertension	1.49	1.04–2.12	**0.02**
Diabetes	0.93	0.67–1.28	0.64
Immunosuppression	0.65	0.36–1.17	0.15

Table S2: Variables associated with discharge alive from the ICU in 322 patients with severe COVID-19, using a cause-specific hazard model, with mortality considered as a competing event.

	**Multivariate**		
**Variables**	Adjusted Hazard Ratio	IC 95	p
**Corticosteroids**	0.73	0.53–0.99	**0.046**
**Male Sex**	0.74	0.53–1.04	**0.082**
**Age**	0.97	0.95–0.99	**0.0004**
Chronic Kidney Disease	1.51	0.83–2.75	0.18
**Hypertension**	1.50	1.06–2.15	**0.02**
Diabetes	0.94	0.67–1.29	0.68
Immunosuppression	0.65	0.36–1.19	0.15

Table S3: Characteristics (pathology and medication) of the thirty patients considered as immunosuppressed.

Patient	Underlying pathology	Immunosuppressive therapy
1	Lymphoma	methotrexate, rituximab
2	Kidney transplant	corticosteroids, tacrolimus, mycophenolate
3	Neutropenia	post chemotherapy
4	Kidney transplant	corticosteroids, tacrolimus, mycophenolate
5	Kidney transplant	corticosteroids, tacrolimus, mycophenolate
6	Ankylosing spondylitis	adalimumab, ledertrexate
7	Lymphoma	rituximab
8	Chronic lymphocyte leukemia, hypogammaglobulinemia	ibrutinib
9	Kidney transplant	corticosteroids, tacrolimus, mycophenolate
10	Systemic lupus erythematosus	corticosteroids, mycophenolate
11	Rheumatoid arthritis	ledertrexate and medrol
12	Kidney transplant	corticosteroids, tacrolimus, mycophenolate
13	Kidney transplant	corticosteroids, tacrolimus, mycophenolate
14	Systemic lupus erythematosus	corticosteroids, mycophenolate
15	Rheumatoid arthritis	methotrexate
16	Lymphoma	post chemotherapy
17	Cardiac transplant	corticosteroids, cyclosporine, mycophenolate
18	Rheumatoid arthritis	etanercept
19	Kidney transplant	corticosteroids, everolimus, mycophenolate
20	Rheumatoid arthritis	corticosteroids
21	Lymphoma, chronic lymphopenia	rituximab
22	Rheumatoid arthritis	ledertrexate
23	HIV	
24	Inflammatory bowel disease	vedolizumab
25	Multiple sclerosis	cladribine
26	Sarcoidosis	corticosteroids
27	Kidney transplant	tacrolimus
28	Rheumatoid arthritis	corticosteroids
29	Myasthenia	corticosteroids
30	Acute myeloid leukemia	corticosteroids, methotrexate

Table S4: Patients characteristics between those developing VA-LRTI or not.

	**All population (n = 322)**	**No VA-LRTI (n = 137)**	**VA-LRTI (n = 185)**	**p value**
**Baseline characteristics**				
Age (+/- SD)	64,8 (+/- 10,4)	64,1 (+/- 10,5)	65,2 (+/- 10,3)	0.30
Male (%)	219/322 (68)	81/137 (59,1)	138/185 (74,6)	0.0033
BMI (+/- SD)	29,7 (+/- 6,2)	30 (+/- 6,2)	29,4 (+/- 6,1)	0.33
Hypertension (%)	204/322 (63,3)	92/137 (67,1)	112/185 (60,5)	0.19
Diabetes mellitus (%)	132/322 (41)	52/137 (38)	80/185 (43,2)	0.37
Cardiomyopathy (%)	49/322 (15,2)	17/137 (12,4)	32/185 (17,3)	0.24
Chronic kidney disease (%)	33/322 (10,2)	14/137 (10,2)	19/185 (10,3)	0.99
Immunosuppression (%)	30/322 (9,3)	14/137 (10,2)	16/185 (8,7)	0.48
COPD (%)	28/322 (8,7)	10/137 (7,3)	18/185 (9,7)	0.46
Neoplasia < 2 years (%)	21/322 (6,5)	6/137 (4,4)	15/185 (8,1)	0.19
**Admission characteristics**				
SOFA (+/- SD)	5,6 (+/- 2,4)	5,9 (+/- 2,5)	5,4 (+/- 2,4)	0.13
APACHE II (+/- SD)	16 (+/- 5,5)	16 (+/- 5,5)	16 (+/- 5,5)	0.95
PaO2/FiO2, mmHg (+/- SD)	93,7 (+/- 47,8)	94,3 (+/- 49,5)	93,2 (+/- 46,7)	0.85
Mild ARDS (PaO2/FiO2 < 300 mmHg)	10/322 (3,1)	5/137 (3,6)	5/185 (2,7)	0.62
Moderate ARDS (PaO2/FiO2 < 200 mmHg)	89/322 (27,6)	33/137 (24)	56/185 (30,3)	0.22
Severe ARDS (Pa02/FiO2 < 100 mmHg)	219/322 (68)	96/137 (70,1)	123/185 (66,5)	0.50
Ferritin, mcg/L (+/- SD), *157 missing values*	2041 (+/-1794)	2260 (+/- 1885)	1922 (+/- 1740)	0.25
CRP, mg/L (+/- SD)	172,9 (+/- 101,5)	169,3 (+/- 103,3)	175,3 (+/- 100,5)	0.61
Lymphocytes, /mcL (+/- SD)	840 (+/- 996)	794 (+/- 496)	873 (+/- 1237)	0.49
Shock (%)	53/322 (16,4)	26/137 (19)	27/185 (51)	0.29
Prior antibiotic treatment (%)	143/322 (44,7)	75/137 (55,2)	68/185 (36,8)	0.0012
**Treatment**				
Hydroxychloroquine (%)	64/322 (19,9)	31/137 (22,6)	33/185 (17,8)	0.27
Azythromycin (%)	19/322 (5,9)	9/137 (6,5)	10/185 (5,4)	0.65
Remdesivir (%)	13/322 (4)	4/137 (2,9)	9/185 (4,9)	0.38

Early corticosteroid (%)	195/322 (60,6)	72/137 (52,6)	123/185 (66,4)	0.011
Immunomodulating therapies, IL6 and IL1 antagonist (%)	12/322 (3,7)	5/137 (3,7)	7/185 (3,8)	0.95
ECMO (%)	44/322 (13,7)	12/137 (8,8)	32/185 (17,3)	0.027
Prone positioning (%)	271/322 (84,2)	127/137 (92)	144/185 (77,8)	0.0040
Sedation, days (+/- SD)	14,4 (+/-13,2)	8,7 (+/- 7,6)	18,5 (+/- 14,8)	< 0.0001
Hospital admission to intubation, days (+/- SD)	4,2 (+/- 5,8)	4,2 (+/- 5,2)	4,2 (+/- 6,3)	0.98

VA-LRTI: ventilator-associated lower respiratory tract infection; ICU: intensive care unit; BMI: body mass index; COPD: chronic obstructive pulmonary disease; SOFA: sequential organ failure assessment; APACHE II: acute physiology and chronic health evaluation II; ARDS: acute respiratory distress syndrome; ECMO: extra-corporeal membrane oxygenation.

Table S5: Patients' outcomes between those developing VA-LRTI or not.

	All population (n = 322)	No VA-LRTI (n = 137)	VA-LRTI (n = 185)	p value
ICU Mortality (%)	151/322 (46,9)	57/137 (41,6)	96/185 (51,9)	0.067
ICU Day 28 mortality (%)	120/322 (37,3)	53/137 (38,7)	67/185 (36,2)	0.65
ICU length of stay, days (+/- SD)	23,1 (+/- 20,9)	15,6 (+/- 15,2)	28,6 (+/- 22,9)	< 0.0001
Duration of ventilation, days (+/- SD)	18,3 (+/- 17)	11,4 (+/- 11,6)	23,3 (+/-18,5)	< 0.0001
Ventilatory-free day D28 (+/- SD)	6,8 (+/- 8,7)	10 (+/- 9,7)	4,4 (+/- 7)	< 0.0001
Bloodstream infection (%)	73/322 (22,6)	16/137 (11,7)	57/185 (30,8)	< 0.0001

VA-LRTI: ventilator-associated lower respiratory tract infection; ICU: intensive care unit.

Table S6: Patients characteristics between first vs. second and third waves.

	All population (n = 322)	First wave (n = 91)	Second and third waves (n = 231)	p value
**Baseline characteristics**				
Age (+/- SD)	64,8 (+/- 10,4)	63,3 (+/- 10,4)	65,3 (+/- 10,3)	0.11
Male (%)	219/322 (68)	60/91 (65,9)	159/231 (68,8)	0.62
BMI (+/- SD)	29,7 (+/- 6,2)	29,3 (+/- 4,8)	29,9 (+/- 6,6)	0.43
Hypertension (%)	204/322 (63,3)	92/137 (67,1)	145/231 (62,8)	0.64
Diabetes mellitus (%)	132/322 (41)	29/91 (32,1)	103/231 (44,6)	0.043
Cardiomyopathy (%)	49/322 (15,2)	9/91 (9,9)	40/231 (17,3)	0.10
Chronic kidney disease (%)	33/322 (10,2)	4/91 (4,3)	29/231 (12,6)	0.032
Immunosuppression (%)	30/322 (9,3)	6/91 (6,5)	25/231 (10,8)	0.25
COPD (%)	28/322 (8,7)	6/91 (6,5)	22/231 (9,5)	0.42
Neoplasia < 2 years (%)	21/322 (6,5)	3/91 (3,2)	18/231 (7,8)	0.051
**Admission characteristics**				
SOFA (+/- SD)	5,6 (+/- 2,4)	5,5 (+/- 2,6)	5,7 (+/- 2,4)	0.71
APACHE II (+/- SD)	16 (+/- 5,5)	15,9 (+/- 5,4)	16 (+/- 5,5)	0.87
PaO2/FiO2, mmHg (+/- SD)	93,7 (+/- 47,8)	100 (+/- 51)	91 (+/- 46,4)	0.11
Mild ARDS (PaO2/FiO2 < 300 mmHg)	10/322 (3,1)	4/91 (4,4)	6/231 (2,6)	0.40
Moderate ARDS (PaO2/FiO2 < 200 mmHg)	89/322 (27,6)	31/91 (34)	58/231 (25,1)	0.10
Severe ARDS (Pa02/FiO2 < 100 mmHg)	219/322 (68)	55/91 (60,4)	164/231 (71)	0.068

Ferritin, mcg/L (+/- SD), *157 missing values*	2041 (+/-1794)	2239 (+/- 2017)	1997 (+/- 1745)	0.50
CRP, mg/L (+/- SD)	172,9 (+/- 101,5)	190,4 (+/- 100)	167 (+/- 102)	0.074
Lymphocytes, /mcL (+/- SD)	840 (+/- 996)	820 (+/- 450)	847 (+/- 1143)	0.83
Shock (%)	53/322 (16,4)	16/91 (17,6)	37/231 (16)	0.73
Prior antibiotic treatment (%)	143/322 (44,7)	62/91 (68,8)	81/231 (35,2)	< 0.0001
**Treatment**				
Hydroxychloroquine (%)	64/322 (19,9)	61/91 (67,8)	3/231 (1,3)	< 0.0001
Azythromycin (%)	19/322 (5,9)	16/91 (17,7)	3/231 (1,3)	< 0.0001
Remdesivir (%)	13/322 (4)	6/91 (6,6)	7/231 (3)	0.14
Early corticosteroid (%)	195/322 (60,6)	3/91 (3,3)	192/231 (83,1)	< 0.0001
Immunomodulating therapies, IL6 and IL1 antagonist (%)	12/322 (3,7)	2/91 (2,2)	10/231 (4,3)	0.36
ECMO (%)	44/322 (13,7)	14/91 (15,4)	30/231 (13)	0.57
Hospital admission to intubation, days (+/- SD)	4,2 (+/- 5,8)	3,7 (+/- 4,3)	4,4 (+/- 6,3)	0.32

VA-LRTI: ventilator-associated lower respiratory tract infection; ICU: intensive care unit; BMI: body mass index; COPD: chronic obstructive pulmonary disease; SOFA: sequential organ failure assessment; APACHE II: acute physiology and chronic health evaluation II; ARDS: acute respiratory distress syndrome; ECMO: extra-corporeal membrane oxygenation.

Table S7: Patients' outcomes between first vs. second and third waves.

	All population (n = 322)	First wave (n = 91)	Second and third waves (n = 231)	p value
ICU Mortality (%)	151/322 (46,9)	35/91 (38,5)	118/231 (51,1)	0.041
Duration of ventilation, days (+/- SD)	18,3 (+/- 17)	18,3 (+/- 13,5)	18,3 (+/-18,2)	0.99
Ventilatory-free day D28 (+/- SD)	6,8 (+/- 8,7)	7,5 (+/- 8,2)	6,5 (+/- 8,9)	0.40
VA-LRTI	185/322 (57,5)	40/91 (44)	145/231 (63)	0.0021
Bloodstream infection (%)	73/322 (22,6)	16/91 (17,6)	57/230 (24,8)	0.17

VA-LRTI: ventilator-associated lower respiratory tract infection; ICU: intensive care unit.
